# PepA and ArgR do not regulate Cre recombination at the bacteriophage P1 *loxP* site

**DOI:** 10.1016/j.plasmid.2007.12.001

**Published:** 2008-03

**Authors:** Alasdair I. MacDonald, Yangjie Lu, Elizabeth A. Kilbride, Aram Akopian, Sean D. Colloms

**Affiliations:** Institute of Biomedical and Life Sciences, Division of Molecular Genetics, University of Glasgow, Anderson College, 56 Dumbarton Road, Glasgow G11 6NU, Scotland, UK

**Keywords:** Plasmid stability, Site-specific recombination, Xer-*cer*, Cre-*lox*, *PepA*, *ArgR*

## Abstract

In the lysogenic state, bacteriophage P1 is maintained as a low copy-number circular plasmid. Site-specific recombination at *loxP* by the phage-encoded Cre protein keeps P1 monomeric, thus helping to ensure stable plasmid inheritance. Two *Escherichia coli* DNA-binding proteins, PepA and ArgR, were recently reported to be necessary for maintenance or establishment of P1 lysogeny. PepA and ArgR bind to regulatory DNA sequences upstream of the ColE1 *cer* recombination site to regulate site-specific recombination by the XerCD recombinases. This recombination keeps ColE1 in a monomeric state and helps to ensure stable plasmid maintenance. It has been suggested that ArgR and PepA play a similar role in P1 maintenance, regulating Cre recombination by binding to DNA sequences upstream of *loxP*. Here, we show that ArgR does not bind to its proposed binding site upstream of *loxP*, and that Cre recombination at *loxP* in its natural P1 context is not affected by PepA and ArgR in vitro. When sequences upstream of *loxP* were mutated to allow ArgR binding, PepA and ArgR still had no effect on Cre recombination. Our results demonstrate that PepA requires specific DNA sequences for binding, and that PepA and ArgR have no direct role in Cre recombination at P1 *loxP*.

Bacteriophage P1 is maintained in lysogens as a low copy-number circular plasmid and has an active partition system that ensures accurate plasmid segregation at cell division (Nordström and Austin, 1989). Homologous recombination between two copies of the prophage produces head-to-tail plasmid dimers that interfere with the control of DNA replication (Park et al., 2001) and could also prevent active plasmid partition. P1 encodes a site-specific recombinase, Cre, which acts at the P1 *loxP* site to resolve any plasmid multimers formed by homologous recombination and helps ensure stable plasmid inheritance (Austin et al., 1981).

High copy-number plasmids also utilise site-specific recombination to maintain themselves in the monomeric state, thereby maximising the number of independently segregating plasmid copies and helping to ensure stable inheritance (Summers and Sherratt, 1984). The naturally occurring plasmid ColE1 contains a site known as *cer*, which is a substrate for the chromosomally encoded Xer site-specific recombination system (Stirling et al., 1988a). Recombination is catalysed by the XerC and XerD recombinases, which cut and rejoin recombining partner sites within a 30 bp core located at one end of *cer* (Blakely et al., 1993; Colloms et al., 1990; Summers et al., 1985). Recombination is regulated by the accessory proteins PepA and ArgR, which bind to approximately 180 bp of accessory sequences adjacent to the *cer* core. This regulation ensures that recombination is exclusively intramolecular so that plasmid multimers are resolved to monomers but not *vice versa* (Alén et al., 1997; Colloms et al., 1996; Summers, 1989).

The Xer recombinases are inactive at the *cer* core in the absence of accessory proteins, and are activated by PepA and ArgR binding to the accessory sequences. Recombination occurs in a nucleoprotein complex in which the accessory sequences of two *cer* sites are wrapped around each other three times (Alén et al., 1997). PepA is the main architectural component of this interwrapped complex and can synapse two *cer* sites even in the absence of ArgR. The synaptic complex forms only between directly repeated sites on the same DNA molecule, and this requirement ensures that recombination is always intramolecular. A consequence of this mechanism is that the product of recombination has a specific topology, consisting of two circular DNA molecules interlocked in a 4-node catenane (Fig. 1A) (Colloms et al., 1997). These interlocked circles can be separated by type II topoisomerases, allowing them to segregate independently at cell division.

In contrast to recombination at *cer*, Cre recombination requires only a simple 34 bp *loxP* core site and the Cre protein (Abremski and Hoess, 1985). Recombination is less topologically constrained than recombination at *cer*, acting on sites in direct and indirect repeat (Abremski and Hoess, 1985) and producing topologically complex as well as simple products (Abremski et al., 1983; Kilbride et al., 1999). Cre recombination appears to be more tightly regulated in vivo than it is in vitro, acting predominantly to resolve plasmid multimers in vivo (Adams et al., 1992). Although Cre can function without any other proteins on a minimal *loxP* site, it is possible that there are regulatory elements beyond the 34 bp *loxP* core site that act in conjunction with accessory proteins to regulate Cre recombination in vivo. This would be analogous to Xer recombination at a class of sites such as *cer3*, which undergo recombination in the absence of accessory proteins and sequences, but which are regulated by these factors when they are present (Guhathakurta et al., 1996).

By placing *loxP* adjacent to appropriate DNA sequences, Cre recombination can be made to form specific topologically complex products (Gourlay and Colloms, 2004; Kilbride et al., 1999). For instance, when *loxP* was placed adjacent to the accessory sequences from *cer*, Cre recombination produced 4-noded catenane in the presence of PepA and ArgR. Thus, Cre recombination can take place within the interwrapped complex formed by PepA, ArgR and *cer* accessory sequences (Gourlay and Colloms, 2004). A mutant form of Cre (CreC1) has been isolated that requires the presence of *cer* accessory sequences and PepA for recombination (Gourlay and Colloms, 2004). This mutant displays topological specificity, recombining sites only if they are in direct repeat and producing exclusively 4-noded catenane.

It was recently reported that stable maintenance of a P1 prophage requires the presence of functional *pepA* and *argR* genes (Paul and Summers, 2004). By analogy with the function of PepA and ArgR at *cer*, and based on the ability of PepA and ArgR to regulate Cre recombination at hybrid *cer*-*loxP* sites, it was postulated that PepA and ArgR might act on sequences present adjacent to *loxP* in P1 to regulate Cre recombination (Paul and Summers, 2004). This regulation would ensure that Cre resolves but does not form plasmid multimers, and would be required for stable P1 inheritance.

ArgR binds to 18 bp Arg-boxes, normally found overlapping promoters of ArgR-regulated genes in the *E. coli* genome (Lim et al., 1987). The *cer* accessory sequences contain a single 18 bp Arg-box, 98 bp upstream of the XerC binding site (Stirling et al., 1988b). A match to the Arg-box consensus sequence was noted by Paul and Summers (2004), 97 bp to one side of *loxP* in the sequence of P1 (Fig. 1B and C). However, no direct evidence was obtained that ArgR binds to this putative Arg-box, or that PepA and ArgR affect P1 stability by regulating Cre/*loxP* recombination. In this study, we investigate the role of PepA and ArgR in Cre recombination, and directly test the hypothesis that these proteins bind to sequences adjacent to *loxP* in P1.

To ascertain whether PepA and ArgR affect Cre recombination at the natural *loxP* site in the same way as they do at the hybrid *cer*-*loxP* site, we carried out in vitro Cre recombination reactions on P1 *loxP* in the presence and absence of PepA and ArgR. To ensure that any P1 accessory sequences required for the proper regulation of Cre recombination were included in our experiments, *loxP* was amplified from P1 together with sequences extending 329 bp to the left of the *loxP* core (towards the *cI* gene) and 236 bp to the right of the *loxP* core (towards the *cre* gene; Fig. 1B). This fragment includes the proposed *loxP* Arg-box, 97 bp to the left of *loxP*. Together with the 34 bp *loxP* core site, this fragment is 601 bp long and is hereafter referred to as *loxP*-601. A recombination substrate (p*loxP*-601), carrying two directly repeated copies of *loxP*-601 separated by a kanamycin resistance cassette was constructed in a high copy-number plasmid vector (Fig. 2). This plasmid substrate was then incubated with purified Cre in the presence and absence of PepA and ArgR. The reaction products were nicked with DNase I and run on an agarose gel to reveal the product topology (Fig. 2B). Control reactions were carried out with p*cer*-*loxP^2^*, which carries two copies of a hybrid *cer*-*loxP* recombination site, consisting of 220 bp of *cer* accessory sequences adjacent to a 34 bp minimal *loxP* site. As previously observed (Gourlay and Colloms, 2004), the addition of PepA changed the topology of Cre recombination at *cer*-*loxP* so that the major product was 4-noded catenane (Fig. 2A). ArgR acted cooperatively with PepA, so that lower concentrations of PepA were required to give the same change in topology (Fig. 2A, compare lanes 2 and 4; and data not shown). ArgR also had an effect on the product topology in the absence of PepA, increasing the amount of 4-noded catenane product (Fig. 2A; compare lanes 3 and 5). In contrast, PepA and ArgR had no effect on recombination at *loxP* in its natural P1 context (Fig. 2B). The major products of recombination, at all concentrations of both PepA and ArgR, were unlinked circles and Holliday junction intermediates, identical to those formed by Cre alone. If PepA and ArgR formed an interwrapped complex with sequences adjacent to the *loxP* site in P1, similar to that formed with *cer* accessory sequences, this would be reflected in a change in product topology in the presence of ArgR and PepA. The absence of topologically complex products in the presence of PepA and ArgR implies that no such interwrapped complex is present.

The lack of topologically complex products could be explained if PepA and ArgR regulate recombination at P1 *loxP* in a complex that does not interwrap the two recombining sites. In this case, PepA and ArgR would still be expected to activate recombination by the PepA-dependent Cre mutant CreC1. To test this, in vitro recombination reactions were carried out with CreC1, in the presence and absence of PepA and ArgR. No recombination was observed with CreC1 at *loxP*-601 in the presence or absence of PepA and ArgR (Fig. 2E). Control reactions with CreC1 on p*cer*-*loxP^2^* confirmed that PepA stimulates recombination by CreC1, giving 4-node catenane as primary product (Fig. 2D), as previously reported (Gourlay and Colloms, 2004). Furthermore, PepA and ArgR had no effect on the amount of recombination catalysed by wild-type Cre on *loxP*-601 in a 30 min reaction (Fig. 2B, compare the amount of unrecombined open circle substrate in lanes 2–5). In contrast, PepA increased the extent of recombination by wild-type Cre on *cer*-*loxP* (Fig. 2A, compare the amount of unrecombined substrate in lanes 2 and 5). Taken together, the above results show that PepA and ArgR do not act on the P1 sequences present in *loxP-601*, either to alter the reaction topology or to stimulate Cre recombination at *loxP*.

Paul and Summers (2004) identified a potential Arg-box by sequence similarity to known Arg-boxes, upstream of *loxP* in a similar position relative to the crossover site as the Arg box in *cer*. This sequence has a 16 out of 18 bp match to the ArgR consensus binding site, but is identical to the *cer* Arg-box at only 8 out of 18 positions (Fig. 1C). To investigate whether this sequence can bind ArgR, a 198 bp DNA fragment containing the proposed Arg-box was amplified from p*loxP*-601 by PCR, labelled with ^32^P and used in a gel mobility shift assay with ArgR. The *loxP* fragment remained almost completely unbound even at the highest ArgR concentrations used (200 nM hexamer; Fig. 3A). In contrast, a DNA fragment containing the *cer* Arg-box was almost completely bound at 100 nM ArgR, and 50% bound at approximately 10 nM ArgR (Fig. 3C). It is not certain why the Arg-box-like sequence upstream of *loxP* fails to bind ArgR, but we note that the A at position 7 and the C at position 10 (Fig. 1C) are both very unusual in known ArgR binding sites.

To investigate what happens when ArgR can bind upstream of *loxP*, we altered the putative Arg-box in *loxP*-601 by site-directed mutagenesis, making it identical to the Arg-box in *cer*. Ten single nucleotide changes were made in *loxP*-601 (Fig. 1C), yielding a DNA fragment here referred to as *loxP*-601^∗∗^. Gel mobility shift assays confirmed that this new Arg-box upstream of *loxP* was efficiently bound by ArgR (Fig. 3B).

Two copies of *loxP*-601^∗∗^ were then placed in direct repeat in a high copy-number plasmid vector to make the recombination substrate p*loxP*-601^∗∗^ (Fig. 2). p*loxP*-601^∗∗^ was then incubated in vitro with Cre or CreC1, in the presence and absence of PepA and ArgR. The products were nicked with DNaseI and separated on an agarose gel. The presence of a functional Arg-box upstream of *loxP* made no difference to the recombination products obtained: p*loxP*-601^∗∗^ gave unlinked circles and Holliday junction intermediates with wild-type Cre in the presence and absence of PepA and ArgR (Fig. 2C), and recombination with CreC1 was not stimulated by PepA and ArgR either alone or in combination (Fig. 2F).

As PepA and ArgR did not regulate recombination by Cre in vitro, we wanted to ascertain whether they are required for the function of Cre/*loxP* in plasmid segregation in vivo. We first tested whether PepA and ArgR are required for P1 replication and active partition in vivo. Two plasmids, pALA136 and pALA1557 (a kind gift from Finbarr Hayes and Stuart Austin), containing functional replication origins from both pBR322 and P1 were used. The pBR322 origin allows for easy maintenance and manipulation of these plasmids in standard *E. coli* strains. When they are introduced into a *polA* mutant strain, where the pBR322 replication origin is not functional, they are maintained at a low copy-number under the control of the P1 replication origin. pALA136 (Martin et al., 1987) lacks any active partition systems, and is therefore not stably maintained in the absence of selection in *polA* mutant strains. pALA1557 (Radnedge et al., 1997) is identical to pALA136 except that it contains the P1 active partition region, which encodes the active partition proteins ParA and ParB and the centromere-like *parS* site on which they act. These two plasmids were tested for stable maintenance in the *polA* mutant strain BR825 (Ludtke et al., 1989), and *argR* and *pepA* mutant derivatives of BR825 made by P1 transduction. Plasmids were introduced into these strains by transformation, and plasmid retention was measured after approximately 20 generations of growth in non-selective liquid culture. As expected, the partition genes greatly increased stable plasmid inheritance of pALA1557 compared to pALA136. Mutations in *argR* and *pepA* had no effect on either plasmid: pALA136 was equally unstable, and pALA1557 was equally stable in all three strains (Fig. 4). Thus, ArgR and PepA are not required for P1 replication or active partition in vivo.

To test the effect of Cre–*loxP* recombination on these plasmids, a 1783 bp DNA fragment containing *cre*, *loxP* and 297 bp to the left of *loxP* (Fig. 1B) was inserted into the unique SalI restriction sites in pALA136 and pALA1557 to create pEK138 and pEK139, respectively. The stable inheritance of these two plasmids was then tested in the *polA* mutant strain BR825 and its *argR* and *pepA* derivatives. There was no significant difference in plasmid retention between BR825 and its *argR* and *pepA* mutant derivatives. However, rather than increasing plasmid stability as expected, the Cre/*loxP* fragment greatly reduced stable maintenance of both pALA136 and pALA1557. The Cre/*loxP* fragment appeared to have a toxic effect on *E. coli* when present on multicopy plasmids, giving reduced growth rates and plasmid yields (data not shown), and it may have a similar effect in pEK138 and pEK139, reducing their apparent stability by reducing the growth rate of plasmid-containing cells.

Because the Cre/*loxP* fragment did not stabilise the mini-P1 plasmids used in this study, we are unable to draw any firm conclusions about the role of PepA and ArgR in this stabilisation in vivo. Nevertheless, the in vitro data argue strongly that PepA and ArgR do not stabilise P1 by regulating Cre recombination at *loxP*. ArgR does not bind to the proposed Arg-box upstream of *loxP* in P1, and therefore cannot act on Cre recombination by binding to this site. PepA and ArgR had no effect on the topology of Cre recombination at *loxP* in its natural P1 context, and failed to stimulate recombination by wild-type Cre or the accessory factor dependent mutant CreC1, even when the putative ArgR binding site was mutated so that it could bind ArgR.

We have recently developed an electrophoretic mobility shift assay in which PepA binds to *cer* accessory sequences cooperatively with ArgR. In the same assay, PepA does not bind to sequences upstream of *loxP*, even when they are altered to allow ArgR binding (Tong, MacDonald and Colloms, unpublished data). These results reinforce the idea that PepA does not act on Cre/*loxP* recombination in P1 and imply that PepA recognises specific sequences that are present in *cer* but absent from *loxP* flanking sequences.

Interestingly, ArgR changed the topology of recombination at *cer*-*loxP* in the absence of PepA (Fig. 2A), but had no effect on recombination at the *loxP*-601^∗∗^ site (Fig. 2B), even though this site contains a functional *cer* Arg-box. The absence of topologically complex products from p*loxP*-601^∗∗^ implies that the presence of an Arg-box at the correct distance from *loxP* is not sufficient to interwrap the two sites. We suggest that ArgR can synapse the Arg-boxes in a pair of *cer* accessory sequences without the need for PepA, and this together with the intrinsic bend in the *cer* accessory sequences (Summers, 1989) can lead to interwrapping of the two sites such that some of the product is 4-node catenane.

Since PepA and ArgR play no direct role in Cre recombination at P1, the loss of P1 stable inheritance in an *argR* mutant, and the inability to form a lysogen in a *pepA* mutant observed by Paul and Summers (2004) must be due to some other effect. To attempt to find a role for ArgR in P1 maintenance, we used an alignment of known Arg-boxes to search the entire P1 genome (Łobocka et al., 2004) for possible Arg-boxes using the EMBOSS programs PROPHECY and PROFIT (Rice et al., 2000). The highest scoring Arg-box sequence is at nucleotide 4375 in P1, within *res*, which encodes a type III restriction enzyme (Hümbelin et al., 1988). The next two top scoring Arg-box sequences are the one upstream of *loxP* at position 94669 and one within the *simC* superinfection exclusion gene (Maillou and Dreiseikelmann, 1990), at nucleotide 47270. Interestingly, there are two adjacent Arg-box sequences within *simC*, separated from each other by just 5 nucleotides, and a third at position 47906, precisely at the junction of the *simA* and *simB* genes. These sequences have not yet been tested for their ability to bind ArgR, and it is not known if they are responsible for the reduced stability of P1 in an *argR* mutant observed by Paul and Summers (2004). Because no useable consensus binding site has been defined for PepA, we have not been able to carry out a similar search for PepA binding sites in P1, and can provide no further insights into the role of PepA in P1 biology.

Finally we note that Cre/*loxP* recombination supports chromosome dimer resolution in *E. coli* in an FtsK-dependent manner (Capiaux et al., 2002). It would therefore be interesting to test whether Cre recombination is also regulated by FtsK on P1.

## Figures and Tables

**Fig. 1:**
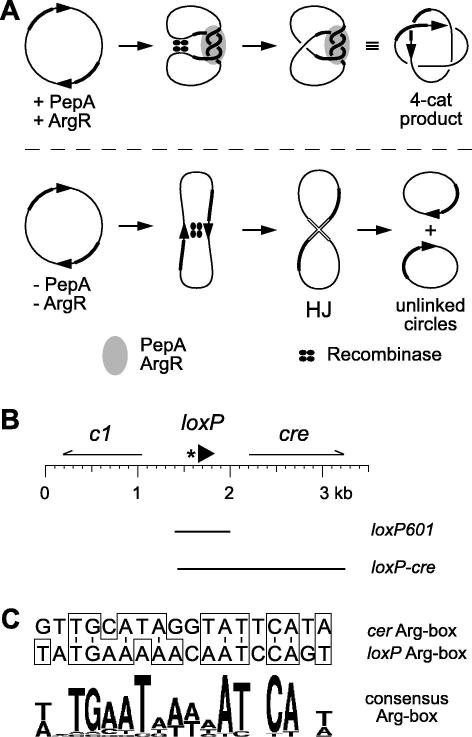
PepA and ArgR in site-specific recombination. (A) Schematic diagram to show how wrapping of accessory sequences around PepA and ArgR alters the topology of recombination. Recombination sites *cer* and *cer*-*loxP* consist of core sites (filled triangles) adjacent to accessory sequences (thick lines). Recombination substrates contain two directly repeated recombination sites. PepA and ArgR bind accessory sequences to form an interwrapped synapse such that the product is a 4-noded catenane (4-cat). In the absence of accessory proteins, the major products are unlinked recombinant circles and “Figure-8” Holliday junction (HJ), an intermediate in the pathway leading to unlinked circles. (B) Map of the bacteriophage P1 *loxP* region. *loxP* is between the genes for the phage repressor (*c1*) and the Cre recombinase. The 34 bp *loxP* core is indicated by a filled triangle. The position of the proposed ArgR binding site 97 bp upstream of *loxP* is indicated by an asterisk. The extents of the *loxP-*601 and the 1783 bp *loxP*–*cre* fragments used in this study are shown below the map. Three short open reading frames (*coi*, *imcB* and *imcA*) are found between *loxP* and *c1*, and another (*cra*) is located between *loxP* and *cre*. (C) Alignment of the Arg-box in *cer*, the proposed Arg-box upstream of *loxP* and the Arg-box consensus generated from an alignment of 17 known ArgR binding sites (Charlier et al., 1992) using the program weblogo (Crooks et al., 2004; Schneider and Stephens, 1990). Identities between P1 and *cer* sequences are shown with vertical lines and matches to the consensus are boxed.

**Fig. 2:**
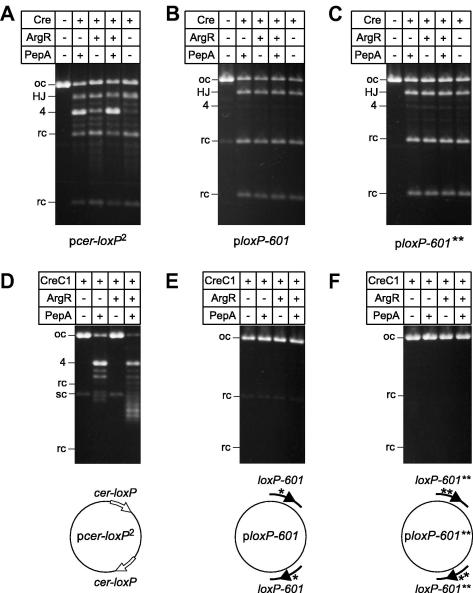
Effect of ArgR and PepA on the topology of Cre recombination. Plasmids containing two directly repeated *cer*-*loxP*, *loxP601* or *loxP601*^∗∗^ recombination sites were incubated with wild-type Cre or the PepA-dependent Cre mutant (CreC1) in the presence and absence of PepA and ArgR as indicated. Reactions were nicked with DNase I and run on agarose gels to display the product topology. Products are indicated as follows: unlinked circular recombination products, rc; 4-noded catenane, 4; Holliday junction intermediate in the production of unlinked circles, HJ; unreacted open circular substrate, oc; supercoiled unreacted substrate DNA, sc. Reactions were carried out for 30 min, as described in Gourlay and Colloms (2004). A small amount of large recombinant circle is present in the p*loxP-601* substrate DNA. Maps of plasmid recombination substrates are shown at the bottom of the Fig. The hybrid *cer*-*loxP* site is shown as an open arrow. The *loxP*-601 and *loxP*-601^∗∗^ fragments are shown as lines with *loxP* as a filled triangle. The putative Arg-box upstream of *loxP* (∗) and this sequence mutated to be identical to the Arg-box in *cer* (∗∗) are indicated.

**Fig. 3:**
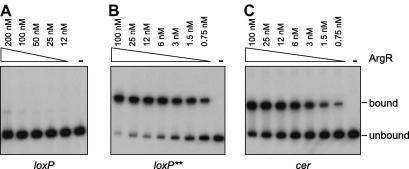
Titration of ArgR binding to *loxP* DNA fragments. ^32^P-labelled 198 bp PCR products from P1, extending from 215 bp to the left of *loxP* to 18 bp to the left of *loxP*, or the same sequence mutated to contain the Arg-box from *cer* (*loxP*^∗∗^), or a 190 bp Arg-box-containing fragment from *cer*, were used in electrophoretic mobility shift assays with purified ArgR. Binding reactions contained the indicated concentration of ArgR hexamer. The positions of bound and unbound DNA fragments are indicated. Binding reactions were carried out in the presence of 1 mM l-arginine essentially as described in Burke et al. (1994) except that the ArgR dilution buffer contained 20 mM Tris-HCl pH 8.2, 20 mM MgCl_2_, 150 mM NaCl, 5 mM dithiothreitol, 1 μg/ml bovine serum albumin and 0.01% Triton X-100. Reactions were run on non-denaturing 5% polyacrylamide gels containing 10% glycerol with 0.33× TBE and 1 mM l-arginine in the gel and running buffer.

**Fig. 4:**
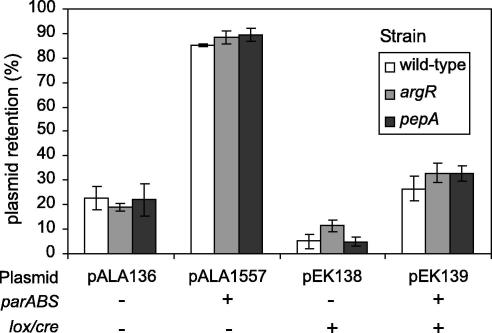
Stability of P1 mini-plasmids in *argR* and *pepA* mutant strains. The indicated plasmids were transformed into the *polA* mutant strains BR825 (wild-type for *pepA* and *argR*), BR825 *argR* and BR825 *pepA*. Plasmids contain the P1 *parABS* active partition cassette and a 1783 bp *loxP*–*cre* cassette as indicated. Transformants were selected for resistance to chloramphenicol and streaked out to single colonies on chloramphenicol-containing plates. For each assay, a single colony was inoculated into Luria Bertani (LB) broth and grown overnight at 37 °C without selection. The overnight culture was diluted and spread onto non-selective LB agar plates to yield single colonies, 50 of which were picked and replica-streaked onto selective and non-selective plates to measure plasmid loss. The graph shows the mean and standard deviation of at least 4 independent assays for each combination of plasmid and strain.
